# γ-Glutamyl Transferase Is an Independent Biomarker of Splanchnic Thrombosis in Patients With Myeloproliferative Neoplasm

**DOI:** 10.1097/MD.0000000000003355

**Published:** 2016-05-20

**Authors:** Jan Görtzen, Lena M. Hunka, Maria Vonnahme, Michael Praktiknjo, Andrea Kaifie, Rolf Fimmers, Christian Jansen, Annkristin Heine, Jennifer Lehmann, Joachim R. Goethert, Norbert Gattermann, Eray Goekkurt, Uwe Platzbecker, Peter Brossart, Christian P. Strassburg, Tim H. Brummendorf, Steffen Koschmieder, Dominik Wolf, Jonel Trebicka

**Affiliations:** From the Department of Internal Medicine I (GJ, HLM, PM, JC, LJ, SC, TJ); Department of Medical Clinic III, University of Bonn, Bonn (VM, HA, BP, WD); Department of Hematology, Oncology, Hemostaseology, and SCT, Faculty of Medicine, University Hospital of the RWTH Aachen University, Aachen (KA, BTH, KS); Department of Biometrics, Informatics and Epidemiology, University of Bonn, Bonn (FR); Department for Hematology, University Hospital Essen, Essen (GJ); Department for Hematology, Oncology and Clinical Immunology, University Hospital Duesseldorf, Duesseldorf (GN); Practice for Hematology-Oncology Eppendorf, Hamburg (GE); and Department for Hematology, University Hospital Dresden, Dresden, Germany (PU).

## Abstract

Myeloproliferative neoplasms (MPNs) are associated with an increased risk of thrombotic events and constitute the major risk factor of splanchnic venous thrombosis (SVT) in Western countries. Although timely anticoagulation resolves SVT, unrecognized SVT frequently leads to portal hypertension and, potentially, variceal bleeding, which may render anticoagulation difficult. Thus, early identification of SVT development is clinically relevant in MPN patients.

In this retrospective analysis, we included 126 patients with MPN and/or SVT referred to our hospital between 2009 and 2014. A total of 86 patients diagnosed with MPN formed the first cohort (PV n = 18, ET n = 16, and MF n = 40), whereas 40 patients who had SVT without adjunct MPN formed a control cohort. Median follow-up period was 960 days. Clinical and laboratory data were collected and analyzed for the identification of potential biomarkers applying descriptive statistics, nonparametric testing, Kaplan–Meier, and logistic regression analysis. The relevance of the identified biomarkers was evaluated in an independent 2nd cohort of 181 patients from the MPN registry of the Study Alliance of Leukemia (SAL-MPN).

Thirty-three MPN patients (38%) in the 1st cohort had SVT. Elevated levels of aspartate aminotransferase, alanine aminotransferase, serum bilirubin, or γ-GT were significantly correlated to the presence of SVT. In multivariate testing, CRP and aspartate aminotransferase were predictors for survival and γ-GT remained the only significant variable associated with SVT in MPN patients (*P* < 0.05). These findings were confirmed in the 2nd cohort comprising 42% of patients with MPN suffering from SVT.

Elevated γ-GT levels indicate SVT in MPN patients, whereas CRP levels are independent predictors of patient survival.

## INTRODUCTION

Myeloproliferative neoplasms (MPNs) represent a heterogeneous group of chronic blood diseases, with clonal hematopoiesis of one or more blood cell lineages leading to altered cellular blood composition and a high risk of venous thromboembolism.^1–3^ Prospective cohort multicenter studies revealed that MPN is a major cause for the development of splanchnic venous thrombosis (SVT).^4–6^ In patients with MPN and SVT, permanent anticoagulation is recommended to prevent further thrombotic events and progression of thrombosis.^7,8^ However, due to unspecific symptoms, SVT is typically underdiagnosed.^8^ In these cases, acute complications such as life-threatening mesenteric ischemia may develop. Chronic SVT (e.g., portal cavernoma) is associated with portal hypertension and its complications such as gastrointestinal variceal bleeding and ascites, as well as hepatic encephalopathy and cholangiopathy.^7,8^ SVT permeation is feasible under anticoagulation in about half of the patients after 6 months of acute SVT, but this is not expected in patients with chronic SVT.^4,7–9^ Therefore, timely diagnosis of SVT and early start of anticoagulation are of paramount importance.^7,8^ Contrast-media enhanced imaging is required to rule out or to assess the presence and extent of SVT, which is too costly and time-consuming to be used as a screening method for every MPN patient. The aim of this retrospective analysis was to identify markers for SVT in MPN patients, using a large MPN patient cohort with and without SVT, a non-MPN SVT control cohort. The data were validated in an independent 2nd cohort.

## PATIENTS AND METHODS

### Patient Cohorts

A total of 126 consecutive patients admitted to our institution with splanchnic vessel thrombosis and/or MPN diagnosed between 2009 and 2014 were included in this retrospective analysis. The ethical committee of the University of Bonn approved the study (EK 254/14) and all patients gave their written informed consent. Routinely evaluated clinical data of all patients were collected by medical personnel during hospital visits. Data collected included medical history, general clinical data, medication, and laboratory parameters (Tables 1 and 2). Patients were then divided into either the 1st MPN-cohort, consisting of MPN patients with and without SVT, or a control cohort formed by SVT patients without MPN. An independent 2nd cohort of 181 consecutive MPN patients predominantly selected for SVT was provided from the MPN registry of the Study Alliance of Leukemia (Tables 1 and 2). The ethical committee of the RWTH Aachen Faculty of Medicine also approved the study (EK 127/12).

**TABLE 1:**
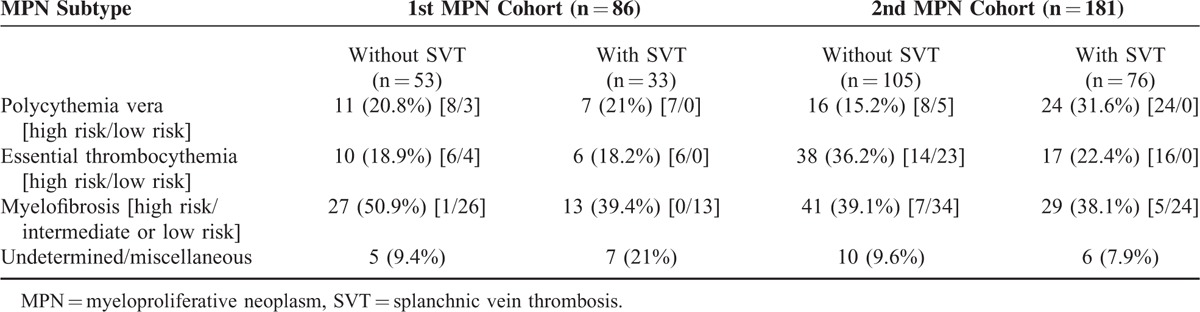
Etiology of MPN in patients With and Without SVT

**TABLE 2:**
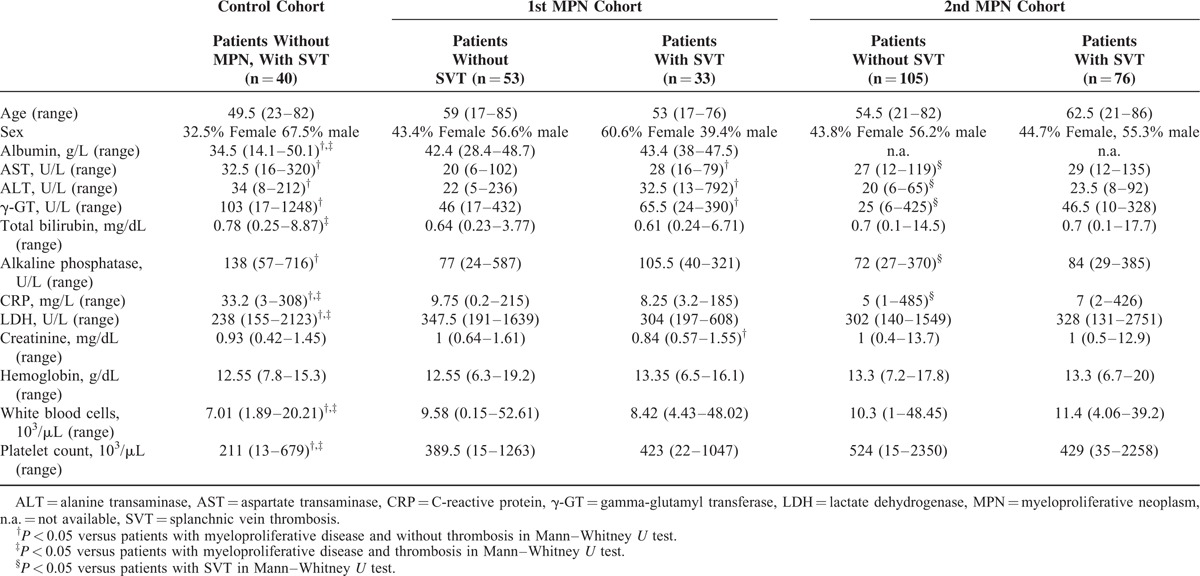
Laboratory Parameters of the Patients in Both MPN Cohorts and the Control Cohort

### Statistical Analysis

Clinical data were collected and evaluated using SPSS statistical analysis software (IBM SPSS Statistics for Windows, Version 22.0, released 2013. Armonk, NY: IBM Corp.). Data of patients with and without MPN were assessed using descriptive statistics. Individual risk for thrombotic complications was stratified using medical history in polycythemia vera (PV) and essential thrombocythemia (ET) patients as described before.^10^ In myelofibrosis (MF) patients, discrimination between low and high risk for survival was estimated using the DIPPS score.^11^ However, DIPPS score calculation was not feasible in the 1st cohort as weight loss, night sweats, and fever were not assessed at inclusion in the study. Mann–Whitney *U* and Kruskal–Wallis tests were used for the comparison of quantitative values between different groups of patients. Receiver operating characteristic (ROC) analysis was performed for parameters that showed significant differences in nonparametric testing. Kaplan–Meier curves were used to analyze either the survival rates or the development rate of SVT, log-rank test was used to compare the time to event between groups. Univariate and multivariate analysis provided information about independent predictors of patient outcome. Logistic regression analysis was used for the multifactorial analysis of predictive variables regarding splanchnic thrombotic events and survival. *P* levels smaller than 0.05 were defined as statistically significant.

## RESULTS

### General Characteristics of Patients Cohorts

A total of 126 patients were included in the 1st MPN and control cohorts. The median age at 1st contact was 53 years (ranging from 17 to 85 years) (Table 2). Baseline demographic variables were similar between both groups, with the exception of non-MPN patients being significantly younger at the initial visit when compared to MPN patients (*P* < 0.05, Table 2). Seven patients died during follow-up, 6 from pneumonia and subsequent sepsis and 1 from hemorrhagic shock following spleen rupture. In the 1st MPN cohort, 18 patients were diagnosed with PV (20.9%), 16 with ET (18.6%), and 40 with MF (46.5%). In 12 patients, the entity of MPN could not be determined (unclassifiable MPN) (12%), as summarized in Table 1. Thirty-four patients were tested for Janus kinase 2 (JAK2) V617F mutation, among which 23 were positive including 22 patients also being diagnosed with MPN according to the WHO diagnostic criteria. One patient was positive for JAK2 V617F mutation without fulfilling the diagnostic criteria for MPN. The 2nd MPN cohort consisted of 181 MPN patients with a median age of 59 years. Forty-two patients had PV (22.2%), 58 patients had ET (30.7%), and 73 patients suffered from MF (42.8%). Seven patients had unclassifiable MPN and 1 patient was diagnosed with platelet-derived growth factor receptor-beta-rearranged MPN (Table 1). MPN patients with SVT were significantly older when compared to those without SVT (62.5 vs 54.5) (Table 2). SVT was present in 76 (40.2%) MPN patients. Twenty-four patients in the 2nd MPN cohort died (12.7%), 11 from disease-related complications, 2 from therapy-related complications, 7 of unknown or undocumented causes, and 4 of other non-related causes.

### Laboratory Parameters in MPN Patients

**FIGURE 1:**
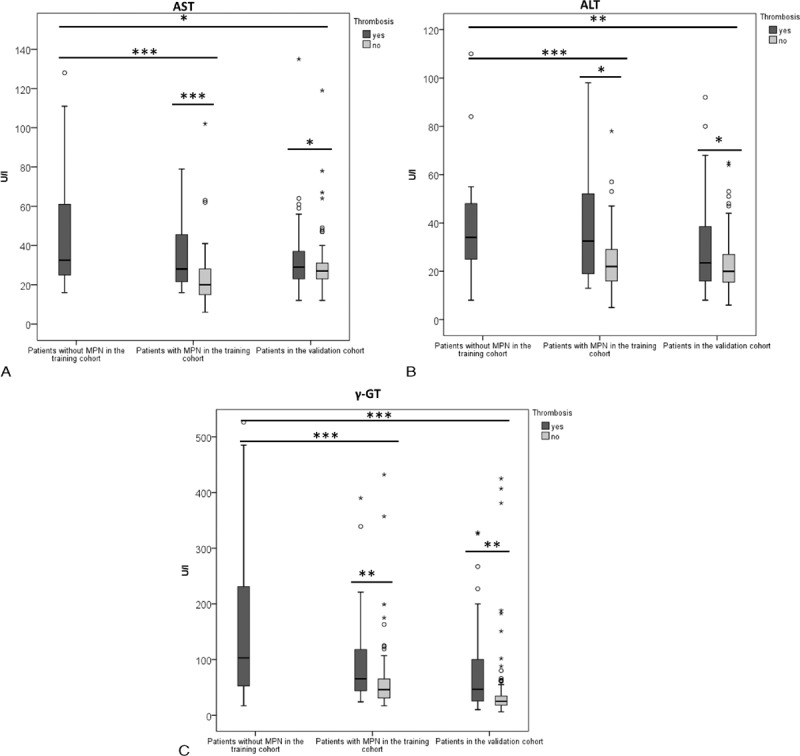
Laboratory parameters of patients with and without MPN. Serum levels of AST, ALT, and γ-GT were significantly higher in patients with non-MPN-associated SVT when compared to MPN patients of the control and 2nd cohort (A–C). Furthermore, MPN patients who had SVT showed significantly higher AST, ALT, and γ-GT levels than MPN patients without SVT in both the control and 2nd cohort. ALT = alanine transaminase, AST = aspartate transaminase, γ-GT = gamma-glutamyl transferase, MPN = myeloproliferative neoplasm, SVT = splanchnic vein thrombosis.

Patients with MPN had significantly higher lactate dehydrogenase (LDH) than patients with non-MPN-associated SVT (*P* < 0.05, Table 2). MPN patients with SVT also showed a trend toward elevated LDH. However, this was not statistically significant (Table 2). Patients with MPN and SVT had significantly higher platelet and white blood cell (WBC) counts but lower C-reactive protein (CRP) levels than those with non-MPN-associated SVT (*P* < 0.01, Table 2). However, this could not be confirmed in the independent 2nd MPN cohort. Patients with non-MPN-associated SVT showed significantly lower serum albumin levels (*P* < 0.01) and higher serum bilirubin levels (*P* < 0.05) when compared to patients with MPN-associated SVT (Table 2). In the 1st MPN cohort, patients with SVT exhibited significant elevation of aspartate aminotransferase, alanine aminotransferase, and gamma-glutamyl transferase (γ-GT) serum levels when compared to MPN patients without SVT. This finding was confirmed in the 2nd cohort (Figure 1A–C, Table 2). Patients with non-MPN associated SVT also had lower serum albumin levels, LDH, and WBC counts than MPN patients (Table 2).

### Risk Factors for SVT

**FIGURE 2:**
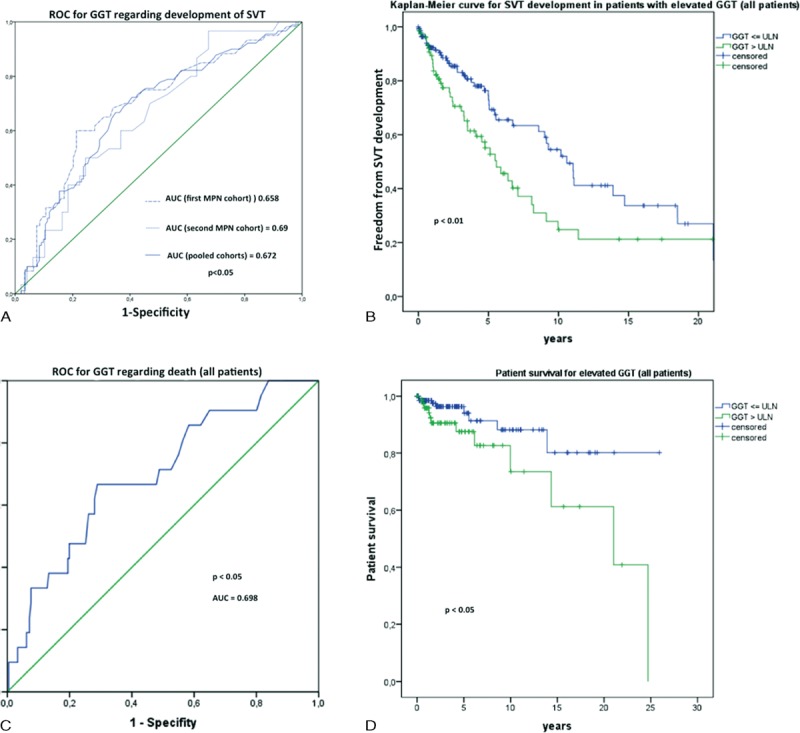
γ-GT as a risk factor for MPN patients. The AUROC was determined to illustrate the predictive values of γ-GT for the presence of SVT in MPN patients (A). In Kaplan–Meier analysis, patients with γ-GT levels higher than the upper limit of normal were significantly more likely to suffer from SVT (*P* < 0.01) (B). Upper limit of normal was defined as 38 U/L for female and 55 U/L for male patients. Furthermore, γ-GT levels were also associated with patient survival (C, D). AUROC = area under the receiver operating characteristic, γ-GT = gamma-glutamyl transferase, MPN = myeloproliferative neoplasm, SVT = splanchnic vein thrombosis.

Interestingly, elevated γ-GT was significantly associated with presence of splanchnic thrombotic events in univariate analysis (Table 3). Area under the receiver operating characteristic (AUROC) was plotted to depict our findings in the 1st cohort, 2nd cohort and all patients combined (Figure 2A). Kaplan–Meier analysis showed significantly higher probability of SVT development in patients who showed elevated γ-GT levels (Figure 2B). Although univariate analysis also suggested elevated serum bilirubin and CRP levels to be risk factors for SVT (*P* < 0.05, respectively), this data could not be confirmed by multivariate analysis. The latter could only confirm elevated γ-GT to be an independent risk factor in all MPN patients to develop SVT (*P* < 0.01, Table 3).

**TABLE 3:**
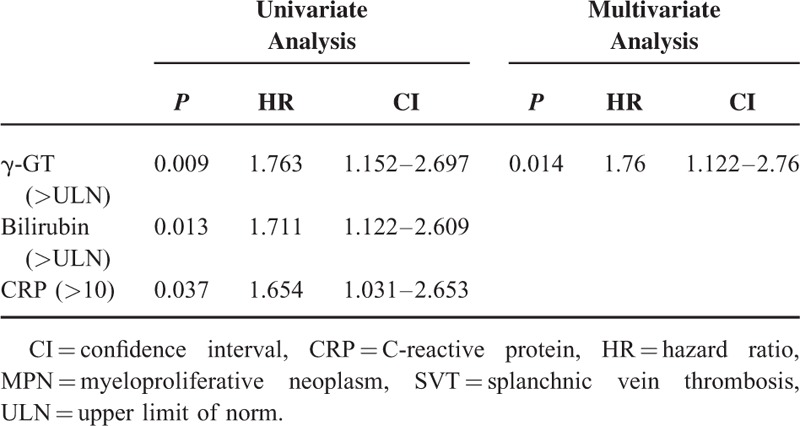
Risk Factors for SVT in MPN Patients

### Risk Factors for Survival

**FIGURE 3:**
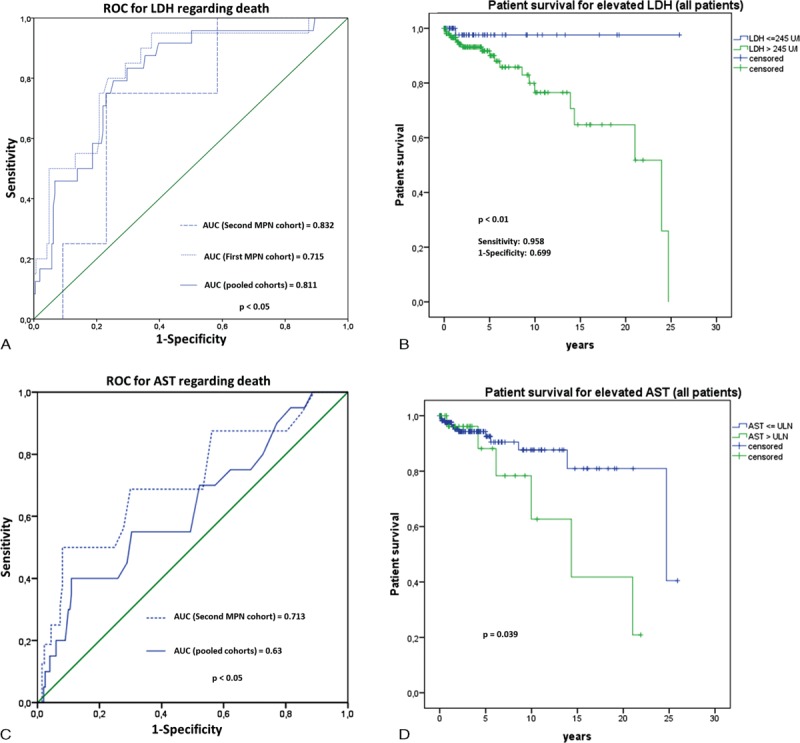
Risk factors for survival in MPN patients. AUROC analysis suggests LDH and AST to be associated with survival in MPN patients (A, C). In Kaplan–Meier analysis, MPN patients with serum LDH higher than 245 U/L had significantly lower cumulative probability of survival (*P* < 0.01) (C). AST levels higher than the upper limit of normal also showed lower survival probability, especially when observed for more than 5 years (*P* < 0.05) (D). Upper limit of normal was defined as 35 U/L for female and 50 U/L for male patients. AST = aspartate transaminase, AUROC = area under the receiver operating characteristic, LDH = lactate dehydrogenase, MPN = myeloproliferative neoplasm.

Similarly, we analyzed the data for factors determining overall survival in MPN patients. Univariate analysis again showed a significant association of elevated levels of LDH (*P* < 0.05), γ-GT (*P* < 0.05), and CRP (*P* < 0.01) with overall survival (Table 4). In AUROC analysis, γ-GT showed again a significant association with patient survival (Figure 2C). In Kaplan–Meier analysis, patient survival was superior in those patients with normal γGT-levels (Figure 2D). Serum LDH and aspartate aminotransferase were also associated with survival in both AUROC and Kaplan–Meier analysis (Figure 3A–D). Last, multivariate analysis shows that only elevated CRP is a risk factor for survival in MPN patients (Table 4).

**TABLE 4:**
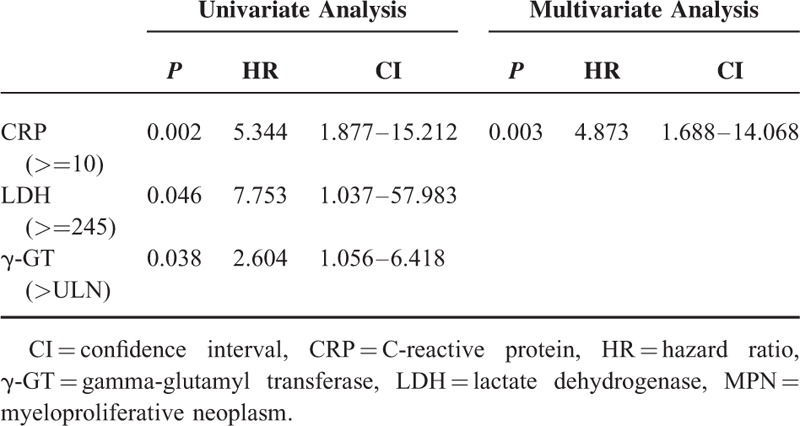
Risk Factors for Death in MPN Patients

## DISCUSSION

The most significant finding of this study is the identification of elevated γ-GT as an independent risk factor for SVT presence in MPN. In addition, we demonstrate that CRP independently correlates with worse overall survival of MPN patients. In a multicenter study including 891 ET patients, the 15-year cumulative risk for thrombosis was 22%.^12^ In another study, thrombotic events were identified as a major cause of death (29%) in PV patients.^13^ As thrombotic events are known to be predictors of mortality and therefore have been included in several models of risk stratification (e.g., international prognostic score of thrombosis in WHO-essential thrombocythemia score), various studies have been conducted to identify predictors of thrombotic events.^14^ Age over 60 years, past history of thrombotic events, and presence of cardiovascular risk factors have been identified to be major determinants for the development of thrombotic events.^15^ More recently JAK2 V617F and Calreticulin (CALR) Exon 9 mutations were found to be associated with a higher incidence of thrombotic events.^16^ However, to date there is no data available on clinical variables predicting SVT in MPN patients. SVT in MPN-patients is a meaningful clinical event, which might lead to either acute and potentially life-threatening intestinal infarction or particularly in the case of unrecognized and untreated SVT, causes potentially severe portal hypertension with a substantial risk of variceal bleeding.^4,7–9^ Vice-versa, in up to 50% of SVT-patients this represents the 1st manifestation of a previously diagnosed MPN.^4–6^ Interestingly, the JAK2 V617F genotype is associated with a frequency of SVT of up to 30%, while the Calreticulin (CALR) Exon 9 mutation is only rarely (1%–2%) associated with SVT.^4–6,17^ Early anticoagulation in SVT is one of the important indicators for the recanalization of the SVT. Recanalization is feasible in about half of the patients after 6 months, but is not expected in patients with chronic SVT.^4,7–9,18,19^ Contrast-media enhanced imaging is recommended to diagnose SVT and evaluate its extent; however, this technology is not useful as screening method. To date there are no biomarkers available that noninvasively establish the diagnosis of SVT in MPN-patients. Our study offers a simple biomarker to better stratify the risk for the development of SVT. γ-GT is universally available and might be part of the routine visits in the out-patient clinics. Abnormal γ-GT is associated with presence of SVT in MPN and non-MPN patients. Therefore, γ-GT might represent a censoring tool for thrombotic events of splanchnic vessels in all patients. Since MPN patients have a high risk of thrombotic events, γ-GT might be used as an easy to assess marker for SVT-screening in these patients. As alkaline phosphatase (AP) and bilirubin were not significantly elevated, our data suggest that elevated γ-GT levels are most likely not originating from cholestasis in these patients. During the period assessed in this study, none of the participating patients of the 1st cohort received any medication or change in the medication that might be associated with an increase or decrease in the levels of γ-GT. Similarly, in the 2nd cohort in none of the patients the increase of γ-GT was suspected to be caused by medication. Therefore, it is unlikely that the association of γ-GT and SVT is influenced by the medication of the patients. With respect to their general characteristics, SVT-patients with and without MPN showed no significant differences in body weight, body mass index, or blood pressure. However, patients who were diagnosed with MPN were significantly older than patients with non-MPN-associated SVT. This suggests that SVT associated with MPNs may have a different pathophysiology as compared to non-MPN-associated SVTs and therefore may be complications during the natural history of the disease. Alternatively, the low incidence of MPN in general may also have led to delayed diagnosis, as the condition is rather uncommon and difficult to detect. As we have shown significantly higher levels of serum bilirubin and lower albumin in non-MPN patients with SVT compared to MPN patients with SVT, it is likely attributed to the fact that other hepatic vascular pathologies involve the liver in noncirrhotic SVT patients. Conversely elevated LDH levels, WBC count, and thrombocytosis in MPN compared to non-MPN patients is likely to be associated with the underlying hematological disease rather than it is a consequence of SVT. This assumption is supported by the multivariate analysis, which identified neither LDH nor WBC or platelet counts as independent SVT predictors in MPN patients. CRP as a marker of chronic inflammation plays a crucial role in development of complications in patients with liver disease,^20^ which at least partly favors the development of SVT.^21,22^ We found this marker to be an independent predictor of mortality possibly highlighting the link between thrombosis and survival in these patients.^13^ However, clinical implications and potential underlying mechanisms require further investigation in the future. The most intriguing result of our study was that γ-GT was clearly elevated in SVT versus non-SVT MPN patients. Univariate and multivariate analysis also implies γ-GT to be a risk factor for the development of SVT in MPN patients. This finding was confirmed in an independent 2nd cohort from the MPN-registry of the Study Alliance of Leukemia. γ-GT is anchored in the cellular membrane, a potential factor leading to increased γ-GT release may be ischemic injury as a consequence of splanchnic vein occlusion. Elevated γ-GT levels in MPN patients are an independent risk factor for SVT. As γ-GT is a routinely evaluated laboratory parameter we postulate that MPN patients with elevated γ-GT levels should be closely monitored for the presence or development of SVT, which can easily been complemented by regular Doppler-ultrasonography of the portal vein before applying a contrast-media-enhanced method. However, to the best of our knowledge, so far there are no biomarkers and no algorithms recommended as screening method for SVT. Our study – despite limitations such as its retrospective nature, registry data in the 2nd cohort, etc. – offers a potential biomarker to screen for SVT in MPN patients and might improve and facilitate the selection of patients requiring imaging and save resources. In conclusion, our study provides 1st evidence that γ-GT levels might screen for SVT in MPN patients. Further and prospective evaluation of the clinical implications are required.
